# Pediatric Orally Disintegrating Tablets (ODTs) with Enhanced Palatability Based on Propranolol HCl Coground with Hydroxypropyl-β-Cyclodextrin

**DOI:** 10.3390/pharmaceutics16111351

**Published:** 2024-10-23

**Authors:** Marzia Cirri, Paola A. Mura, Francesca Maestrelli, Simona Benedetti, Susanna Buratti

**Affiliations:** 1 Department of Chemistry Ugo Schiff (DICUS), University of Florence, 50019 Sesto Fiorentino, Italy; paola.mura@unifi.it (P.A.M.); francesca.maestrelli@unifi.it (F.M.); 2 Department of Food, Environmental and Nutritional Sciences (DeFENS), University of Milan, 20133 Milan, Italy; simona.benedetti@unimi.it (S.B.); susanna.buratti@unimi.it (S.B.)

**Keywords:** pediatric formulation, propranolol HCl, cyclodextrin complexation, orally disintegrating tablets, taste masking, electronic tongue

## Abstract

Background: Propranolol, largely prescribed as an antihypertensive and antiarrhythmic drug in pediatrics, is characterized by a bitter taste and an astringent aftertaste. Currently, the therapy requires crushing of tablets for adults and their dispersion in water many times a day, leading to loss of dosing accuracy, low palatability, and poor compliance for both patients and caregivers. Objectives: This work aimed to exploit cyclodextrin complexation by cogrinding to develop orally disintegrating tablets (ODTs) endowed with reliable dosing accuracy, good palatability and safety, ease of swallowability, and ultimately better compliance for both pediatric patients and caregivers. Results: Different formulation variables and process parameters were evaluated in preparing ODTs. The technological and morphological characterization and disintegration tests were performed according to official and alternative tests to select the ODT formulation based on the drug Hydroxypropyl-β-cyclodextrin (HPβCD) coground complex form containing Pearlitol^®^ Flash as the diluent and 8% Explotab^®^ as the superdisintegrant, which demonstrated the highest % drug dissolution in simulated saliva and acceptable in vitro palatability assessed by the electronic tongue, confirming the good taste-masking power of HPβCD towards propranolol. Conclusions: Such a new dosage form of propranolol could represent a valid alternative to the common extemporaneous preparations, overcoming the lack of solid formulations of propranolol intended for pediatric use.

## 1. Introduction

The development of age-appropriate formulations for children is the main issue of pediatric therapy to assure that all children, of different ages, as well as their caregivers, can easily access to safe and accurate dosage forms, as pointed out by the implementation of pediatric-specific guidelines provided by the Food and Drug Administration (FDA) and the European Medicine Agency (EMA) [1].

Most drugs prescribed for the prevention or treatment of diseases in pediatrics are unlicensed or off-label from the point of view of formulation, therapeutic use, and/or route of administration, with the majority designed for and studied in adults [2].

The lack of suitable specific formulations often leads to a large use of extemporaneous preparations such as crushing of tablets dispensed as suspensions or dilution of existing liquid dosage forms. Such manipulations cause problems of loss of dosing accuracy, unknown bioavailability, and poor compliance for both young patients and their caregivers [3].

Some essential features are required by an optimal pediatric formulation to adequately improve drug acceptability, adherence, and more efficient therapy, such as ease of administration, appropriate dosage form for different pediatric age groups, low frequency of dosing, safety of excipients, palatability, and minimal discomfort or burden to minimize impact on lifestyle.

Among the different kinds of solid oral dosage forms, orally disintegrating tablets (ODTs) can represent a potential promising technological strategy for oral administration of drugs in pediatrics [4,5,6].

They combine all the advantages of solid dosage forms, such as accurate dosing, good stability and compliance of patients, easy manufacturing, and small packaging size with those of liquid formulations, including easier administration with no risks of choking. ODTs can be easily administered to children without requiring hospitalization or the support of medical professionals, and they have been shown to be well-tolerated and safe [7].

ODTs are able to quickly disintegrate inside the mouth, leading to partial drug absorption through the oral mucosa and thus allowing to partially avoid the first-pass metabolism and increase bioavailability [8,9]. On the other hand, their fast disintegration in the oral cavity leads to early contact of the drug with the taste buds, and therefore the palatability degree becomes critical for the patient acceptance [4,10,11].

Taste masking represents a very important property to be considered in the case of ODTs, since the bitter taste of a drug is one of the most significant drawbacks that can cause a reduction in drug adherence and then treatment failure [12]. Among the different possible taste-masking techniques, the use of cyclodextrins could represent an effective, safe, and low-cost strategy due to their capacity to form inclusion complexes with a wide variety of drugs, thus enabling not only the improvement of their physicochemical properties such as solubility and/or stability [13,14], but also to hide their unpleasant taste, as highlighted from a recent review [15]. The favorable effect of cyclodextrin complexation in hindering the bad, bitter taste sensation of the solubilized drugs has been shown, for example, in the case of cetirizine tablets [16], oral dispersible films and buccal films of indomethacin and furosemide [17], or phenylbutyrate ODTs [18,19]. Moreover, orally administered cyclodextrins have an outstanding safety profile, and may be considered safe and valuable excipients also in the pediatric field [20].

Among the different techniques proposed to prepare inclusion complexes with cyclodextrins in the solid state, mechanochemical activation by grinding seems to be a simple, fast, highly efficient, convenient, versatile, sustainable, eco-friendly, and solvent-free method [21].

Propranolol, largely prescribed in pediatrics as an antihypertensive and antiarrhythmic drug [22,23], is characterized by a bitter taste and an astringent aftertaste.

A previous study showed the effectiveness of complexation with hydroxypropyl-β-cyclodextrin in taste masking of liquid dosage forms of propranolol, making it possible to avoid the need to add potentially harmful excipients as sweeteners or flavoring agents and their solvents, commonly used to improve palatability [24].

Currently, there are no marketed solid dosage forms of propranolol specific for children, thus requiring crushing and dispersion in water of fractions of tablets for adults, with the consequent series of drawbacks as above described. Moreover, some excipients used in adult formulations could be potentially toxic for children [25,26,27].

On the basis of all these considerations, it was considered worthy of interest to investigate the feasibility of developing an oral propranolol-based solid dosage form specific for pediatric therapy, endowed with reliable dosing accuracy, high safety in use, good palatability, ease of swallowability, and ultimately better compliance by both pediatric patients and caregivers, by exploiting the approach of ODTs in combination with the use of drug–cyclodextrin complexation for achieving a safe taste masking.

This study, which represents a continuation of our previous one [24], is in line with the current trends in the pediatric field to switch from liquid to novel suitable solid oral dosage forms, mainly due to their easier transport, minor stability and storage issues, and greater accuracy in dosing [28].

Based on the results of our previous study, hydroxypropyl-β-cyclodextrin (HPβCD) was selected as a complexing agent for propranolol, and the equimolar drug:HPβCD coground system was used to prepare ODTs by direct compression. Different formulation variables, such as type and number of excipients, e.g., diluents and superdisintegrants (SDs), as well as process parameters, such as compression time, were investigated. The resulting tablets were characterized in terms of technological and morphological properties according to USP 43 and 11th Ph. Eur. official tests. Alternative tests to the USP and Ph. Eur. disintegration test specifically developed for ODTs, such as the simulated wetting test (SWT) and test on wire cloth [29,30,31], were also performed. Finally, the best ODT formulations were selected for dissolution studies through the flow-cell dissolution apparatus and for in vitro palatability evaluation by the electronic tongue, which emerged as a valid instrument for a rapid preliminary taste evaluation and screening of oral dosage forms [32,33] in comparison with analogous placebo and free-drug-loaded ODT formulations.

## 2. Materials and Methods

### 2.1. Materials

Propranolol HCl was purchased from Fagron Italia Srl (Quarto Inferiore, Bologna, Italy), hydroxypropyl-β-cyclodextrin (HPβCD) (Cavasol W7 HP) was donated from Wacker Chemie AG (Munich, Germany), Pearlitol^®^ Flash (co-processed mixture of D-mannitol-extra white maize starch) was kindly supplied by Roquette (Lestrem, France), as well as Ludiflash^®^ (co-processed mixture of D-mannitol, crospovidone, polyvinylacetate, povidone) by BASF Pharma (Ludwigshafen, Germany), Pharmaburst^®^ 500 (co-processed mixture of mannitol, silicon dioxide, sorbitol, crospovidone) by SPI Pharma (Wilmington, DE, USA), and Explotab^®^ (sodium starch glycolate) by JRS Pharma, (Rosenberg, Germany). Sodium stearyl fumarate was kindly donated by Menarini Manufacturing (Florence, Italy). Simulated saliva (SS) fluid (8.00 g/L of NaCl, 0.19 g/L of KH_2_PO_4_, and 2.38 g/L of Na_2_HPO_4_) was prepared according to Late et al. [34].

### 2.2. Preparation of Drug:HPβCD Binary Systems

The equimolar drug:HPβCD physical mixture (P.M.), was obtained by gently blending the drug with the carrier for 15 min. The coground product (GR) was prepared by grinding the resulting P.M. in a high-energy vibrational micromill (MM200, Retsch, Haan, Germany) at a frequency of 24 Hz for 30 min.

### 2.3. Drug Assay by UV-Vis Spectrometry

The drug assay was performed by UV spectrometry at λ = 289.6 nm (UV 1601 Shimadzu Europa, Duisburg, Germany). The UV method was validated following the ICH guidance Q2 (R2) [35]. A seven-point calibration curve was performed in a concentration range from 10 to 55 mg/L, obtaining the linear regression equation y = 0.0195x − 0.0065 (R^2^ = 0.9998). The accuracy and precision of the method were also verified by performing three replication measurements on three known concentrations. The resulting LOQ and LOD values were 5.50 mg/L and 1.65 mg/L, respectively.

### 2.4. Preparation of ODTs

ODTs were prepared by direct compression using a single punch Perkin Elmer hydraulic press at a 0.5 ton compression force. Tablets containing 10 mg propranolol HCl as such or as an equimolar physical mixture (P.M.) or coground (GR) with HPβCD were realized. This dosage (10 mg) was selected as a model of pediatric drug dosage, considering the weight-depending recommended doses in children [23]. ODTs at different drug dosages can be produced once the formulation is optimized. Placebo ODTs were also prepared. Pearlitol^®^ Flash, Ludiflash^®^, and Pharmaburst^®^ 500 were tested as coprocessed direct-compression excipients, whereas Explotab^®^ as a superdisintegrant (SD). Na stearyl fumarate was used as a lubricant. All the ingredients of each batch were weighed, and then, except for the lubricant, were blended with a Turbula mixer for 8 min at 50 rpm; then, the lubricant was added and the mixture was blended for a further 2 min. These blending conditions were selected on the basis of preliminary experiments, which proved they were suitable for obtaining mixtures with good homogeneity.

### 2.5. Characterization of ODT Formulations

The prepared ODTs were characterized in terms of weight uniformity, diameter, thickness, hardness (resistance to crushing), and friability according to official pharmacopeia tests.

The disintegration test was conducted by the 11th Ph. Eur. official test and by different specific ODT disintegration tests proposed as an alternative to the official one.

#### 2.5.1. Disintegration Test According to 11th Ph. Eur.

The test was performed on 6 tablets for each batch by a disintegration tester (Erweka, Langen, Germany), using 900 mL of distilled water maintained at 37 ± 0.5 °C. The values were expressed as the average. Times of 3 min or less are required for a tablet to be labeled as ODT, according to the Ph. Eur [36].

#### 2.5.2. Simulated Wetting Test (SWT)

The simulated wetting test (SWT) was performed according to the method proposed by Park et al. to simulate the physiological conditions in the mouth [29]. Briefly, a filter paper disc was placed inside a flat bottom dish (diameter 22 mm) followed by the addition of a suitable volume of simulated saliva (SS) depending on the tablet weight (1.25 mL for tablets from 100 to 379 mg). The ODT was then put on the wet paper so that the liquid only covered the bottom of the tablet. The SWT time, namely the time necessary for the liquid to diffuse through the tablet and fully cover its surface, was determined. The test was performed in triplicate for each batch.

#### 2.5.3. Disintegration Test on Wire Cloth

The “Disintegration test on wire cloth”, developed by Motohiro et al. [31], was also conducted. According to this method, the ODT was placed on a wire cloth (mesh 2 × 2 mm), and simulated saliva (SS) was dropped on it at a constant rate of 4 mL/min [37]. The time required by the tablet to completely pass through the wire cloth was taken as the disintegration time. The test was performed on three tablets for each batch.

### 2.6. Dissolution Test

Drug dissolution behavior from ODTs was evaluated by using the flow-through cell dissolution apparatus (USP apparatus 4) (SOTAX CH-4123, Sotax AG, Aesch BL, Switzerland). A ruby bead (5 mm diameter) was placed at the bottom of a conical dissolution cell filled with glass beads (1 g). A glass filter with pore sizes of 0.5 μm was placed at the outlet of the cell. An ODT was then placed on the glass bead layer. The dissolution medium was simulated saliva (to mimic the conditions in the oral cavity) maintained at 37 ± 0.5 °C and pumped at a flow rate of 4 mL/min; samples were collected every 30 s up to a total time of 3 min, and the drug concentration was measured by UV–spectrometry, as described above (see Section 2.3), after having verified that the presence of excipients did not interfere in the propranolol spectrophotometric assay. The simulated saliva was then replaced by a pH 1.2 solution simulating the gastric environment (40 mL HCl 0.1 N, 0.35 g NaCl, 0.5 g glycine up to 1 L with deionized water). At fixed intervals times (up to a total time of 30 min), samples were collected and assayed by UV–spectrometry (Shimadzu 1601 UV/Vis spectrophotometer, Tokyo, Japan). Experiments were performed in triplicate and the results, expressed as percent of dissolved drug vs time, were averaged.

### 2.7. Scanning Electron Microscopy (SEM)

The samples were analyzed using the Scanning Electron Microscope Gaia 3 (Tescan Brno s.r.o, Brno, Czech Republic) FIB-SEM (Focused Ion Beam-Scanning Electron Microscope). The electron beam used for SEM imaging had a voltage of 10 kV operating in high-vacuum mode and with a secondary electron (SE) detector. Samples were deposited on a stub and coated with an ultrathin coating of silver.

### 2.8. Electronic Tongue Test (E-Tongue)

The analyses were performed with the commercially available e-tongue Taste-Sensing System SA 402B (Intelligent Sensor Technology Co., Ltd., Atsugi, Kanagawa Prefecture, Japan). The detecting part of the system consists of potentiometric sensors whose surface is attached with artificial lipid membranes having different response properties to chemical compounds on the basis of their taste. In this work, four detecting sensors and two reference electrodes (Ag/AgCl) were applied. The detecting sensors, separated into two arrays according to the membrane charge, were specific for the evaluation of bitterness (AC0-bitterness 1 and AN0-bitterness 2), aftertaste bitterness (C00), and aftertaste astringency (AE1) in pharmaceutical formulations.

E-tongue measurements were performed on ODTs containing the drug (10 mg) as such or as coground (GR) with HPβCD, their corresponding ODT placebo (with or without HPβCD), and the pure drug. A proper amount of the sample (10 mg of pure propranolol and 1 tablet for ODT samples) was dissolved in the minimum volume of pure water (20 mL), stirred for 1 min, filtered, and then suitably diluted for e-tongue analysis. The measuring process was the same as reported by Cilurzo et al. [38]. Each sample was evaluated in triplicate and the averages of the sensor outputs were converted to “taste intensity” values by multiplying sensor outputs for appropriate coefficients based on Weber–Fechner law, which provides the intensity of sensation based on the sensor properties for tastes as reported by Kobayashi et al. [39]. E-tongue data were elaborated by Principal Component Analysis (PCA) [40] performed in a covariance matrix at a scale that was the same for all the e-tongue sensors [41]. Data were elaborated by the Minitab 17 software package (v. 1.0, Minitab Inc., State College, PA, USA).

## 3. Results and Discussion

Preliminary studies aimed to select the most suitable conditions to obtain ODT formulations (200 mg, 8 mm diameter) with suitable hardness, and wetting time values were performed. Three different coprocessed direct-compression excipients were tested (all at a fixed amount of 95% *w*/*w*) due to their diluent, filler/binder capacities, as well as disintegrant properties: Pearlitol^®^ Flash, a combination of mannitol and maize starch; Ludiflash^®^, based on mannitol, crospovidone, and polyvinyl acetate; and Pharmaburst^®^ 500, made of mannitol, crospovidone, sorbitol, and precipitated silicon dioxide. They are all high-functionality, ready-to-use excipient combinations especially engineered to reduce manufacturing complexity, allowing to produce, by direct compression, quickly disintegrating tablets having good mechanical features [42].

Sodium stearyl fumarate was added as a lubricant (5% *w*/*w*). The effect of the type of coprocessed excipient, as well as of different compression times (0.5 tons for 10 and 5 s) on hardness and disintegration properties according to the simulated wetting test time (SWT time) [29] selected as key quality parameters of ODT, was evaluated.

Currently, both USP 43 and 11th Ph. Eur. do not describe a disintegration test specific for ODTs, and the test for conventional tablets, owing to both the large volume of medium (900 mL) and the stirring conditions, does not reflect the conditions in the oral cavity, thus making it challenging to effectively evaluate the actual performance of ODT formulations. For this reason, various ODT disintegration methods more properly mimicking the oral conditions have been proposed as an alternative to the official pharmacopeia ones [30,36].

The tablets’ composition and the results of their technological characterization are summarized in Table 1.

Regardless of the type of diluent, the 5 s compression time was enough to obtain tablets with suitable hardness for ODTs, with values around 8–9 Kg, considered enough to provide adequate breakage resistance to crashing without slowing down the disintegration process [8], and shorter SWT time values, considered an indicator of faster disintegration on the tongue. In particular, among the tested diluents, Pearlitol^®^ Flash showed the best performance, giving rise to tablets with the lowest hardness and the shortest SWT time.

The incorporation of 10 mg of the drug, by replacing the same amount of diluent (5% *w*/*w*), did not affect the hardness or SWT time of tablets obtained by 5 s compression time, giving rise to similar results. Therefore, Pearlitol^®^ Flash was selected as an excipient of a new series of tablets (200 mg, 8 mm diameter) containing the drug (10 mg) as such or as a GR system with HPβCD (by replacing the corresponding amount of diluent, i.e., 5% *w/w* for the drug and 30% *w*/*w* for GR) and sodium stearyl fumarate as a lubricant. The effect of the presence of an increasing amount of Explotab^®^ (8 and 16% *w*/*w*) as SD was investigated as well as the effect of different compression times (5, 3, or 1 s).

All the tablets were characterized in terms of technological properties. All the ODT formulations complied with the pharmacopeia requirements for uniformity of weight (RSD < 2%), diameter (RSD < 0.3%), thickness (RSD < 0.6%), and friability (weight loss < 0.4%). In particular, this last value is considered indicative of a suitable abrasion resistance of the tablets. The results of hardness and disintegration properties (performed according to the ‘SWT-time’ and ‘wire cloth’ and the 11th Ph. Eur. official disintegration test), which are considered key quality parameters for ODTs, are shown in detail in Table 2.

As it can be observed, all the tablets loaded with the pure drug exhibited typical ODT properties, showing hardness values ranging from 6 to 9 Kg, disintegration times <3 min according to both the official test and the alternative wire cloth test, and SWT times <30 s, thus satisfying not only the requirements by Ph. Eur. definition but also meeting the more restrictive parameters by FDA guidelines [43].

The hardness was not practically affected by increasing the SD content, whereas a slight increase in the SWT time values was observed especially at the highest % SD, less evident with decreasing the compression time to 1 s.

**Figure 1:**
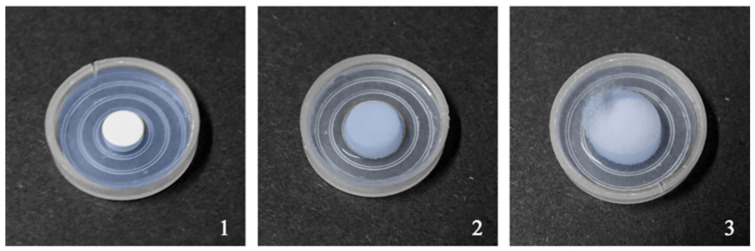
Simulated wetting test of drug-loaded ODT. (**1**): ODT (4A/7A) before testing; (**2**): swelling of ODT 4A (8% SD) at the end of the test (21 s); (**3**): swelling of ODT 7A (16% SD) at the end of the test (28 s).

The slight increase in SWT time with increasing % Explotab^®^ could be explained by the fact that the SD acts by drawing liquid into the tablet through a capillary action, thus resulting in secondary swelling, interparticle bond breakage, and tablet disintegration due to its porous particle morphology. The greater the SD content, the greater the liquid amount absorbed and the longer the time required to expand in volume and swell before disintegration [44], as can be observed in Figure 1.

On the contrary, the introduction of the drug as GR with HPβCD caused a marked increase in the tablet hardness and, consequently, of the SWT times and disintegration time. This was probably due to the high compacting properties of the CD, which caused a reduction in the network porosity required for quick water absorption and consequent fast tablet disintegration. A similar effect has been observed by Commey et al. [19], who reported an about 6-times increase in ODT hardness and a 7-times increase in disintegration time when replacing the drug with its complex with βCD, even using a reduced compression force.

Although the compression time was progressively shortened from 5 s to 3 s up to 1 s, in order to increase the network porosity, being inversely related to the compression time and the exerted pressure, it was not possible to achieve better results.

Furthermore, a reduction in the percentage of CD in the mixture, obtained by increasing the total tablet weight up to 300 mg, thus allowing the introduction of a higher amount of Pearlitol^®^ Flash and the decrease in GR content from 30% to 20% *w*/*w* of the total tablet weight, was not enough to solve the problem, since the tablets showed similar values of both hardness (13–14 Kg), SWT time (>30 s), and disintegration times (>3 min) compared to those of the corresponding tablets weighing 200 mg.

Thus, none of the developed formulations containing the GR drug:HPβCD could be classified as ODT either according to USP or Ph. Eur. Probably, the compression force distributed in the relatively limited tablet surface area, combined with the presence of the CD, gave rise in all cases to a very compact structure of the tablets.

**Figure 2:**
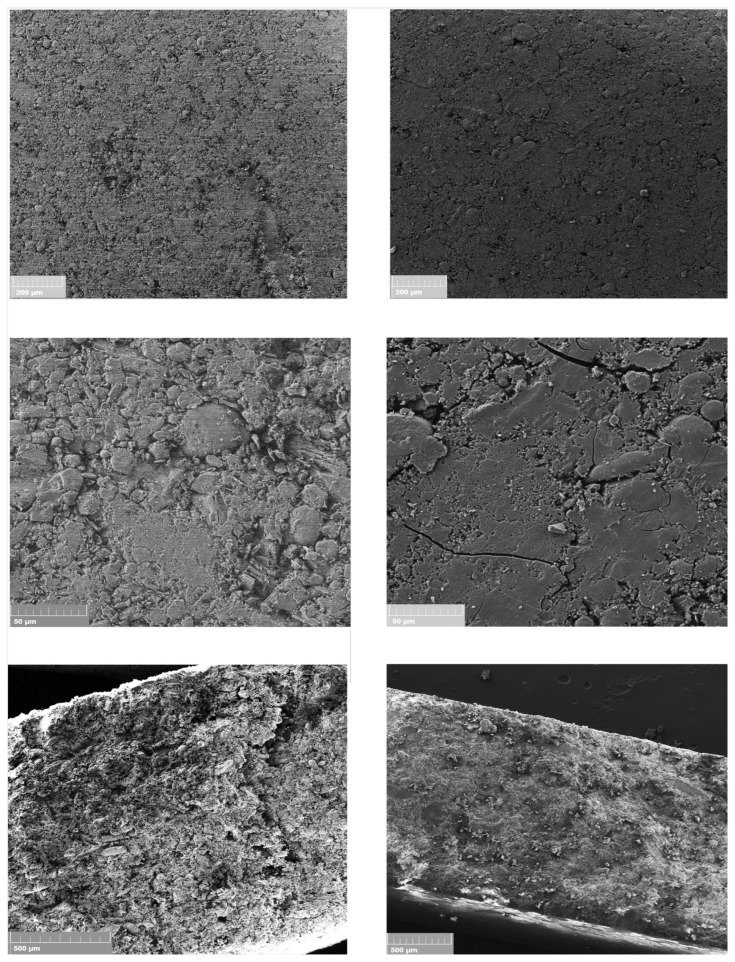
SEM micrographs of selected ODT formulations (9-A, (**left**) and 9-B, (**right**)) of tablet surfaces at different magnifications (**up**) and their cross-section (**down**).

In order to gain insight into the structure of the tablets, SEM analyses were performed on the formulations 9-A and 9-B, containing the drug as such or as GR with HPβCD. Figure 2 shows the SEM images of the surface and the cross-section of the selected tablets at different magnifications.

As can be seen, the tablet containing the drug:HPβCD GR system showed a more compact and smoother surface compared to the tablet containing the pure drug, which had a certain degree of unevenness, also displaying some cracks. The cross-section images clearly showed that the tablet with the binary GR system had a more homogeneous and compact structure compared to that without the GR, which appeared very grainy and porous. These findings confirmed our hypothesis: ODTs containing the pure drug, in virtue of their flaked structure, facilitated the saliva penetration into the tablet, thus leading to a fast disintegration, whereas the more compact structure of the tablets containing the drug:CD GR system hindered and slowed down the whole process.

Therefore, in a further effort to limit the decrease in tablet porosity of the last series of tablets, keeping both the compression time at 1 s and the total tablet weight at 300 mg unchanged, the tablet diameter was increased from 8 to 13 mm in order to distribute the compression force over a wider surface. As can be observed in Table 3, all the tablets met the pharmacopeia requirements for weight uniformity and friability. Such a variation in diameter also allowed for obtaining tablets with lower hardness values compared to their corresponding 8 mm diameter tablets and disintegration times <3 min, thus complying with the Ph. Eur. definition of ODT. These findings showed that the obtained decrease in compression force for the surface area unit was actually able to reduce the compacting effect due to the CD presence, thus leading to both a reduced hardness and an increased porosity of the compact and then to a faster water absorption with a consequent shorter disintegration time.

**Figure 3:**
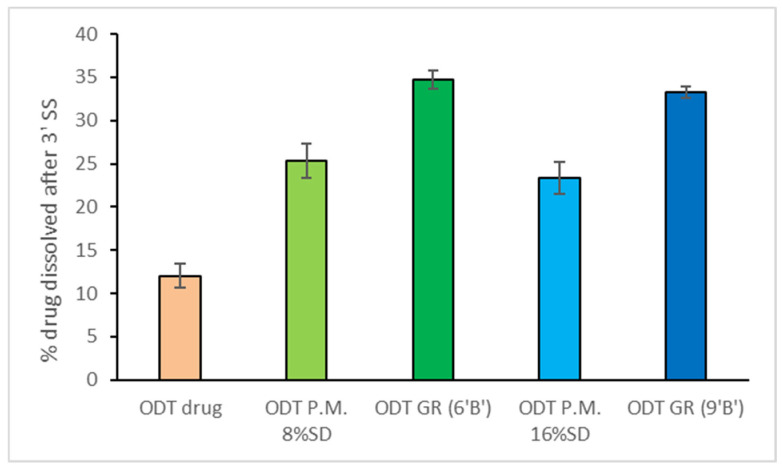
% Drug dissolution in simulated saliva at 37 °C of ODTs containing pure propranolol (ODT drug) or drug:HPβCD as P.M. with 8% SD (ODT P.M. 8% SD) and 16% SD (ODT P.M. 16% SD), or drug:HPβCD as GR with 8% SD (ODT GR 6′ B′) and 16% SD (ODT GR 9′ B′).

This latter series of tablets was tested for drug dissolution properties in simulated saliva at 37 °C, in comparison to analogous tablets containing the drug as such. The progress of drug dissolution was evaluated for 3 min, the maximum estimated time for the tablet permanence in the mouth. Tablets containing the drug as a simple physical mixture (P.M.) with HPβCD were also tested for comparison purposes. As can be observed in Figure 3, ODTs loaded with the simple P.M. showed a higher dissolution rate than those containing the drug alone, confirming the favorable role of the simple presence of the CD in improving the drug dissolution behavior, in virtue of its hydrophilic and wetting properties. On the other hand, ODTs loaded with the GR system showed a clearly better performance than the corresponding ones loaded with the P.M., exceeding the 30% drug dissolved after 3 min, indicating the effectiveness of the co-grinding technique in promoting drug–CD interactions and complex formation, thus further improving the drug dissolution properties, and then, hopefully, as a consequence, its bioavailability. In fact, the favorable effect of using drug–CD interaction products for formulating ODTs with enhanced dissolution and bioavailability of other poorly soluble drugs such as eslicarbazepine [45] or vardenafil [46] has been reported.

No further appreciable improvements in the drug dissolved amount after 3 min in simulated saliva (maximum permanence time in the mouth) were obtained by doubling the % SD from 8 to 16%. Then, the ODT formulation loaded with the drug as GR with HPβCD and containing 8% Explotab^®^ (ODT 6′ B′, Table 3) was selected for further dissolution studies performed up to 30 min, replacing after 3 min the simulated saliva with pH 1.2. solution simulating the gastric medium. A complete drug dissolution was obtained after only 20 min in the case of ODT 6′ B′, while the ODT containing the pure drug, taken as a reference, reached only about 50% dissolved drug after 30 min (see Figure S1).

### Palatability Studies by E-Tongue

E-tongue analysis was finally performed to investigate the palatability of the selected ODT formulation containing the drug as GR with HPβCD (ODT 6′ B′, Table 3) in order to evaluate the actual taste-masking ability of the CD; for this purpose, analogous ODT formulations containing the drug as such (ODT drug), the corresponding ODT placebos, with and without HPβCD, and the pure drug at the same dosage (10 mg) were also tested.

**Figure 4:**
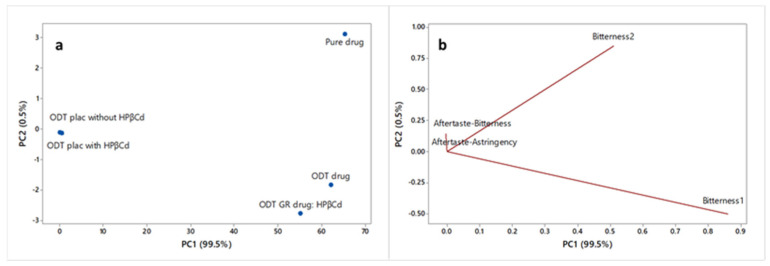
PCA score plot (**a**) and loading plot (**b**) of the collected e-tongue data.

The obtained data were elaborated by Principal Component Analysis (PCA) in order to achieve a partial visualization of the data set in a reduced dimension. Figure 4 reports the score plot (Figure 4a), representing the relationship among samples, and the loading plot (Figure 4b), showing the relationship among variables, in the plane defined by the first two principal components (PC1 and PC2), explaining the total variability (100% of the total variance).

Considering the score plot (Figure 4a), a clear separation of the samples along the PC1 is evident (99.5% explained variance). In particular, placebo samples (ODT placebos with and without HPβCD), located to the left of PC1, are well discriminated from the pure drug and from the two tested formulations (ODT drug; ODT GR drug:HPβCD (6′ B′)), located on the opposite side of the plot. According to the loading plot (Figure 4b), it can be observed that the two bitterness sensors (bitterness 1 and bitterness 2) are significant in the discrimination of the samples, while the aftertaste bitterness and aftertaste astringency are not relevant.

Since the bitter taste is the only relevant one, for all the analyzed samples, the intensity of bitterness 1, bitterness 2, and total bitterness (bitterness 1 + bitterness 2) was considered, and the “bitterness scores” were calculated by setting the bitter intensity of placebos (negative controls) to 0 and the bitter intensity of pure drug (positive control) to 10; the bitterness scores of the two ODT formulations were calculated by the Equation

Bitterness Score = [(BIform − BIplac)/BIdrug] × 10

where BIform is the bitter intensity of each ODT formulation (ODT drug; ODT GR drug:HPβCD), BIplac is the bitter intensity of the corresponding placebo (ODT plac with and without HPβCD), and BIdrug is the bitter intensity of the pure drug.

**Figure 5:**
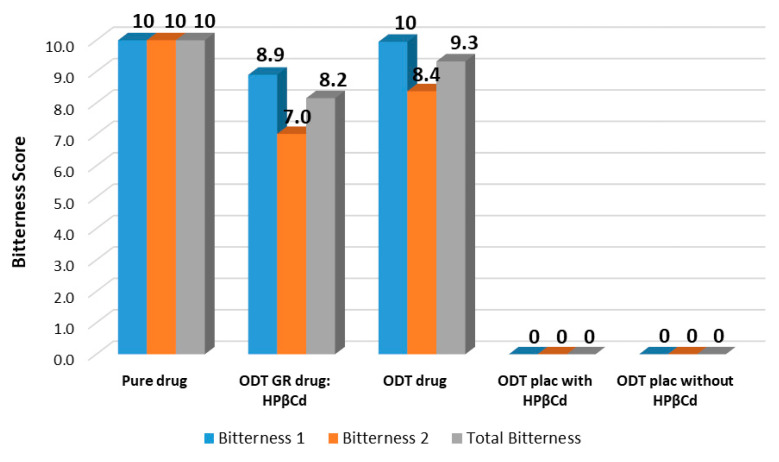
Bar graph of the bitterness scores of pure drug, ODT formulations, and ODT placebos.

Figure 5 shows the bar graph showing the scores for bitterness 1, bitterness 2, and total bitterness (bitterness 1 + bitterness 2) for each sample.

Considering the ODT formulation containing the drug as such (ODT drug), the total bitterness score (bitterness 1 + 2) was equal to 9.3, corresponding to a bitterness reduction percentage of 7% with respect to the pure drug; for the ODT containing the drug as GR with HPβCD (ODT GR drug:HPβCD (6′ B′)), the total bitterness score (bitterness 1 + 2) was 8.2, corresponding to a more marked bitterness reduction percentage (of about 18%) with respect to the pure drug.

Such results confirmed the good taste-masking power of HPβCD towards propranolol, in good agreement with our previous study [24], which has to be attributed to the CD’s ability to encapsulate the drug into its hydrophobic cavity, thus avoiding its direct contact with the taste buds located on the tongue [47,48].

The slight degree of drug bitterness reduction observed for ODT containing the pure drug compared to the powder of the drug alone could probably be ascribed to the presence among the excipients of Pearlitol^®^ Flash, widely used to mask the unpleasant taste of drugs due to the sweetening power of mannitol (similar to that of sucrose), constituting about 80% of this co-processed excipient [49,50].

## 4. Conclusions

In this study, a pediatric ODT formulation of propranolol.HCl endowed with proper technological properties and complying with the requirements of the Ph. Eur. definition was realized for the first time.

The objective was successfully achieved by properly selecting the most suitable excipients and process parameters for direct compression and using the drug in the form of a complex with HPβCD obtained by the safe and effective technique of cogrinding.

The use of propranolol HCl as a complex with HPβCD allowed not only for the improvement of its solubility and dissolution rate, but also reducing its unpleasant bitter taste.

Such a new dosage form of propranolol seemed to be suitable to overcome the lack of solid pediatric formulations of this drug and could represent a valid alternative to the common use of extemporaneous preparations due to its numerous benefits such as high dosing accuracy, ease of administration, and good palatability, which all translate into better compliance by both pediatric patients and caregivers.

The developed formulation could be suited to be produced not only at the industrial level, but also in hospital pharmacies due to the low-cost and easy preparation method, requiring very simple equipment.

Further studies have been planned to test the developed ODT formulation of propranolol HCl in a pediatric hospital, aimed at evaluating its actual clinical efficacy and compliance in pediatric patients.

## Figures and Tables

**Table 1:** Composition of ODTs (200 mg, 8 mm diameter) containing different types of diluents and their properties in terms of hardness and disintegration time determined by the SWT and Ph. Eur test as a function of different compression times.

Code	Compression Time (s)	Pearlitol^®^ Flash (% *w/w*)	Ludiflash^®^ (% *w/w*)	Pharmaburst^®^ 500 (% *w/w*)	Na Stearyl Fumarate (% *w/w*)	Hardness (Kg)	SWT Time (s)	Disint. Time (min)
I	10	95	-	-	5	9 ± 1	31 ± 3	<3
II	10	-	95	-	5	9.5 ± 0.5	42 ± 2	<3
III	10	-	-	95	5	10 ± 1	45 ± 4	<3
IV	5	95	-	-	5	8.5 ± 0.5	16 ± 2	<3
V	5	-	95	-	5	9 ± 0.5	25 ± 2	<3
VI	5	-	-	95	5	9 ± 0	28 ± 3	<3

**Table 2:** Technological properties of 200 mg ODTs (8 mm diameter) containing the drug (10 mg) as such or as coground product (GR) with HPβCD.

Code	Explotab^®^ (%)	Pure Drug (x) or GR	Compression Time (s)	Hardness (Kg)	SWT Time (s)	Wire Cloth Time (min)	Disintegration Time (min)
1-A	-	x	5	9 ± 0.5	18 ± 1	<3	<3
2-A	-	x	3	8 ± 0.5	17 ± 2	<3	<3
3-A	-	x	1	7 ± 0	14 ± 2	<3	<3
1-B	-	GR	5	13 ± 1	>30	>3	>3
2-B	-	GR	3	13 ± 1	>30	>3	>3
3-B	-	GR	1	13 ± 1	>30	>3	>3
4-A	8	x	5	9 ± 0.5	21 ± 0	<3	<3
5-A	8	x	3	8 ± 0.5	18 ± 1	<3	<3
6-A	8	x	1	7 ± 0.5	16 ± 1	<3	<3
4-B	8	GR	5	14 ± 0.5	>30	>3	>3
5-B	8	GR	3	14 ± 0.5	>30	>3	>3
6-B	8	GR	1	13 ± 0.5	>30	>3	>3
7-A	16	x	5	8 ± 0.5	28 ± 2	<3	<3
8-A	16	x	3	7 ± 1	22 ± 0	<3	<3
9-A	16	x	1	6 ± 0	18 ± 2	<3	<3
7-B	16	GR	5	14 ± 0	>30	>3	>3
8-B	16	GR	3	14 ± 1	>30	>3	>3
9-B	16	GR	1	14 ± 0.5	>30	>3	>3

**Table 3:** Properties of 300 mg/13 mm diameter ODTs containing the drug (10 mg) as coground product with HPβCD at different % *w*/*w* Explotab^®^.

Code	Explotab^®^(%)	Compression Time (s)	Hardness (Kg)	SWT Time (s)	Disintegration Time (min)	Weight (mg ± s.d.)	Friability %
6′ B′	8	1	7	>30	<3	301.5 ± 0.9	0.7
9′ B′	16	1	6	>30	<3	300.8 ± 1.2	0.85

## Data Availability

Data are contained within the article and Supplementary Materials.

## Supplementary Materials

The following supporting information can be downloaded at: https://www.mdpi.com/article/10.3390/pharmaceutics16111351/s1, Figure S1: Dissolution curves of propranolol HCl from ODTs containing the drug as such (ODT drug) or as drug:HPβCd GR (ODT 6′ B′) in simulated gastric medium (pH 1.2 solution), after 3 min exposure in simulated saliva.
