# Supplementary material for: Pediatric Orally Disintegrating Tablets (ODTs) with Enhanced Palatability Based on Propranolol HCl Coground with Hydroxypropyl-β-Cyclodextrin
Source: Pharmaceutics. 2024 Oct 23;16(11):1351. doi: 10.3390/pharmaceutics16111351 (PMC11597579; doi:10.3390/pharmaceutics16111351)
Supplement: Supplementary file 1 [file pharmaceutics-16-01351-s001.zip › pharmaceutics-3219976-supplementary.pdf]

pharmaceutics

MDPI

Article

Supplementary Materials: Pediatric Orally Disintegrating Tablets (ODTs) with Enhanced Palatability Based on Propranolol HCl Coground with Hydroxypropyl-β-Cyclodextrin

Marzia Cirri, Paola A. Mura, Francesca Maestrelli, Simona Benedetti and Susanna Buratti

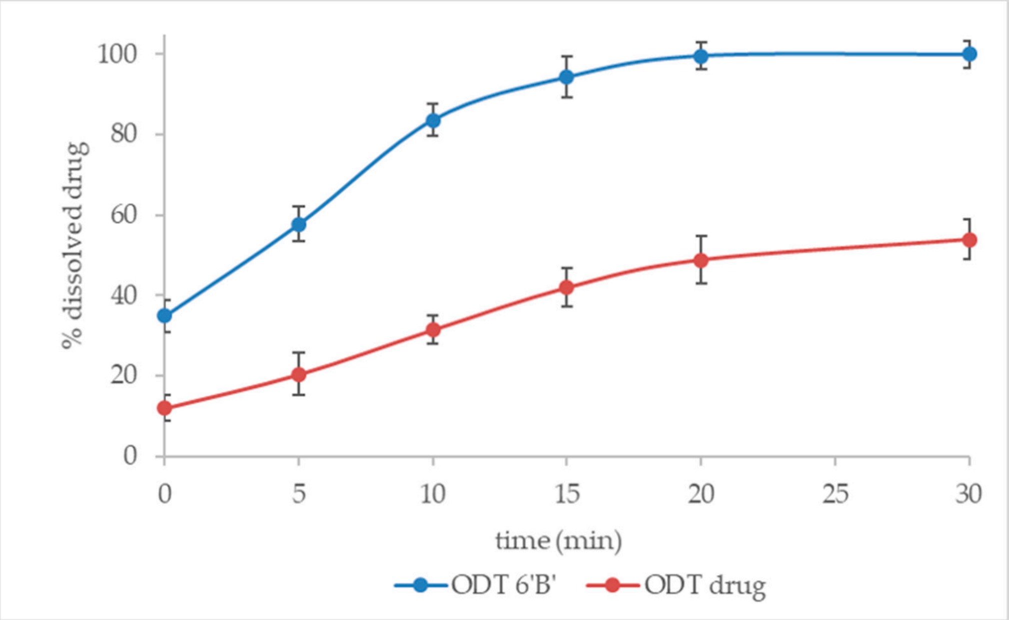

Figure S1. Dissolution curves of propranolol.HCl from ODTs containing the drug as such (ODT drug) or as drug:HPβCd GR (ODT 6'B') in simulated gastric medium (pH 1.2 solution), after 3 min exposure in simulated saliva.
